# Postprandial Metabolism is Impaired in Overweight Normoglycemic Young Adults without Family History of Diabetes

**DOI:** 10.1038/s41598-019-57257-2

**Published:** 2020-01-15

**Authors:** A. Aneesh Kumar, Gopika Satheesh, Gadadharan Vijayakumar, Mahesh Chandran, Priya R. Prabhu, Leena Simon, Vellappillil Raman Kutty, Chandrasekharan C. Kartha, Abdul Jaleel

**Affiliations:** 1 Rajiv Gandhi Centre for Biotechnology, Thiruvananthapuram, Kerala India; 2 Medical Trust Hospital and Diabetes Care Centre, Kulanada, Pathanamthitta, Kerala India; 3 Achutha Menon Centre for Health Science Studies, SCTIMST, Thiruvananthapuram, Kerala India; 4 Manipal Academy of Higher Education, Manipal, Karnataka 576104 India; 5 Society for Continuing Medical Education & Research, Kerala Institute for Medical Sciences, Thiruvananthapuram, 695029 India

**Keywords:** Metabolomics, Endocrine system and metabolic diseases

## Abstract

While the risk factors for Type 2 diabetes (T2DM) are known, early predictive markers of transition from normal to a prediabetes state are unidentified. We studied the basal metabolism and metabolic response to a mixed-meal challenge in 110 healthy subjects in the age group of 18 to 40 years (Male:Female = 1:1); grouped into first degree relatives of patients with T2DM (n = 30), those with a body mass index >23 kg/m^2^ but <30 kg/m^2^ (n = 30), those with prediabetes (n = 20) and normal controls (n = 30). We performed an untargeted metabolomics analysis of plasma and related that with clinical and biochemical parameters, markers of inflammation, and insulin sensitivity. Similar to prediabetes subjects, overweight subjects had insulin resistance and significantly elevated levels of C-peptide, adiponectin and glucagon and lower level of ghrelin. Metabolites such as MG(22:2(13Z, 16Z)/0:0/0:0) and LysoPC (15:0) were reduced in overweight and prediabetes subjects. Insulin sensitivity was significantly lower in men. Fasting levels of uric acid, xanthine, and glycochenodeoxycholic-3-glucuronide were elevated in men. However, both lysophospholipids and antioxidant defense metabolites were higher in women. Impaired postprandial metabolism and insulin sensitivity in overweight normoglycemic young adults indicates a risk of developing hyperglycemia. Our results also indicate a higher risk of diabetes in young men.

## Introduction

Type 2 Diabetes mellitus (T2DM) is a progressive disease characterized by insulin resistance and a relative or absolute deficiency of insulin production. T2DM is diagnosed by elevations in fasting and postprandial levels of glucose and hemoglobin A1C(HbA1c) in blood^1^. Insulin resistance is the early sign towards the onset of T2DM in most of the individuals. Risk factors such as obesity, physical inactivity, high fat-high calorie diet, tobacco, and more than moderate alcohol consumption can worsen an underlying genetic susceptibility for T2DM in insulin-resistant subjects^2^. The response of β-cells to insulin resistance is to increase insulin secretion to maintain normal glucose levels, resulting in hyperinsulinemia. In individuals in whom hyperinsulinemia is ineffective to maintain normoglycemia, fasting glucose and glucose tolerance are impaired. Impaired fasting glucose (IFG) and impaired glucose tolerance (IGT) denote a prediabetes condition, considered as an intermediate stage in the progression of T2DM^3^. Decreased β-cell function is a crucial determinant of transition from prediabetes to T2DM. Genetic abnormalities, glucotoxicity, lipotoxicity, inflammation, and accumulation of amyloid account for impaired β-cell function^1^. Compromised lipid and glucose metabolism in the liver also contributes to the pathogenesis of T2DM^4^. We speculate that the metabolic changes related to T2DM occur earlier in susceptible individuals and could be identified in them before the manifestation of IFG and IGT.

Metabolomics is one of the appropriate system biology tools that have been used to explore the loss of flexibility in metabolism during the onset of T2DM. Metabolomics, employing high-throughput analytical methods is useful to identify and quantify hundreds of metabolites and thus offers an approach not only to discover biomarkers for T2DM and but also to increase our understanding of the process of disease development^5^. In a recent study in individuals with normal fasting blood glucose, information from a discrete set of 19 metabolites was found to improve prediction of T2DM.This study also identified nitrogen metabolism pathway and its components as important in the pathogenesis of T2DM^6^. An earlier Framingham offspring study of patients with new onset of T2DM that used a targeted metabolomic approach observed that plasma levels of five branched-chain amino acids; leucine, isoleucine, tyrosine, valine, and phenylalanine could be useful to predict diabetes. A combination of any three of these amino acids increases the certainty of early diabetes prediction^7^. Metabolic profiling of fasting plasma samples from individuals with varying whole-body insulin sensitivity has revealed that perturbations in fatty acid and amino acid metabolism are associated with T2DM. A reduction in the levels of leucine, dihydrosphingosine, and phytosphingosine were associated with insulin sensitivity^8^. An untargeted metabolomics method was used by Swedish investigators in a nested case-control study in a cohort of patients with new onset of T2DM.They discovered that plasma metabolites phosphatidylcholines (C19:1 and C17:0) and hydroxyethane sulfonate were associated with T2DM^9^. However, there is paucity of metabolomics-based studies on metabolism in healthy people who are at risk for developing T2DM. The objectives of our study were to explore metabolic changes in healthy young adults with known risk factors such as family history and overweight and thus susceptible to develop T2DM and identify early metabolic changes if any in them.

## Methods

### Experimental design

There were 110 study subjects in the age group of 18 to 40 years and an equal number of men and women. Individuals without any illness, including diabetes or any nutritional disorders, were only included for the study. Those on a weight reduction program, with a history of allergy or under medication were excluded from the study. Participants were grouped into: (1) normal healthy controls – NC [n = 30], (2) first-degree relatives of patients with T2DM – FDR [n = 30], (3) those who were overweight – OW [n = 30], and (4) those with prediabetes who served as positive controls – PRD [n = 20].

Participants in the FDR group had either two first-degree relatives or one first-degree and two second-degree relatives with T2DM.Only those with a normal body mass index (BMI less than 23 kg/m^2^) were recruited for the FDR group. Participants in NC and FDR group were matched for their age, sex, and BMI.

Those without having a family history of T2DM and with BMI greater than 23 kg/m^2^ but less than 30 kg/m^2^ were included in the OW group. They were matched with NC for their age and sex. Prediabetes individuals were selected by their impaired fasting glycemia (IFG, fasting glucose level = 100–125 mg/dL) measured on two occasions. The prediabetes group was sex-matched with NC but not for age. Study participants were asked to avoid any unusual activity and intentional exercise during the three days leading up to the initiation of the study. They were also instructed to continue their regular diet without any deviations.

At present, no standard methods are available for the estimation of sample size in untargeted metabolomics analysis and the conventional method of power analysis could not be applied here^10^. In practice, factors including economic and ethical restrictions decide the sample size in metabolomic studies. We have selected 30 study participants in a group based on previous studies^11,12^.

On the study day morning, fasting blood sample was collected from the hand vein of the study participant. Later, a mixed-meal was given based on the calorie required for the ideal body weight of the participant^13^. Ideal body weight was estimated using Lorenz-formula for the calculation of required calories^13^. Calories allotted per kilogram ideal body weight are as follows- Sedentary, 30 kcal/kg body weight; Moderate, 35 kcal/kg body weight; and Heavy, 40 kcal/kg body weight^14^. We prepared a diet such that total calories were distributed in three proximate principles of the diet, such as carbohydrates (55%), fats (30%), and protein (15%). A mixed-meal (Breakfast) with 25% of the total kilocalories required per day, according to Recommended Dietary Allowances (RDA) for Indians (ICMR, 2010) was used for the tolerance test in these study subjects^14^. The calorie content of each food item was calculated from the data for Indian foods, published by the National Institute of Nutrition, Indian Council of Medical Research^15^. A full description of the nutrient composition of the meal is provided in the Supplementary Table S1. Blood samples were collected every 30 minutes until 120 minutes (min) after the meal. Blood samples (6 mL) were collected using vacutainer tubes (BD Vacutainer plus plastic K2 EDTA tubes). The samples were then centrifuged at 2000 × g for 20 min at 4 °C to get plasma and plasma were aliquoted (0.5 mL) and stored at −80 °C until further analysis.

### Measurement of clinical and biochemical parameters

Weight was measured by a digital scale in light clothing and without shoes. A wall-mounted stadiometer was used for the measurement of height. BMI was computed using the weight in kilograms divided by the square of height in meters. For waist-hip ratio (WHR), waist circumference was measured at the midpoint between the lower costal margin and the iliac crest at mid respiration. Blood samples were subjected to biochemical analysis for Glucose, Insulin, Hemoglobin A1c (HbA1c), Serum Total Cholesterol (T.CHOL), Low-Density Lipoprotein-Cholesterol (LDL), High-Density Lipoprotein-Cholesterol (HDL), Triglycerides (TGL), Very Low-Density Lipoprotein-Cholesterol (VLDL), and C-reactive protein (CRP). Plasma glucose was measured by enzymatic hexokinase procedure and T.CHOL, TGL, HDL, LDL, and VLDL were measured by enzymatic methods (AU480 Chemistry System, Beckman Coulter). C-reactive protein was measured by a chemiluminescence immunoassay (Cobas E411 Immunoassay Analyzer, Roche). HbA1c was measured using high-performance liquid chromatography (D10, Bio-Rad Laboratories).

### Insulin sensitivity and beta-cell function

Indices using glucose and insulin values both at fasting and post-meal were utilized to determine insulin sensitivity and beta-cell function. For the fasting-based measurement, Homeostasis Model of Insulin Resistance, HOMA-IR [($Glucos{e}_{0minute}$ × 0.0551 × $Insuli{n}_{0minute}$) ÷ 22.5], was calculated. Oral Glucose Insulin Sensitivity Index(OGIS)^16^, a method based on the meal-stimulated glycemia and insulinemia levels was estimated as done by others^17^.

### Markers of inflammation and T2DM

Plasma levels of obesity/T2DM related markers [Interleukin 6 (IL-6), C-peptide, Glucagon, Insulin, Leptin, Plasminogen Activator Inhibitor-1 (PAI-1), Resistin, Visfatin, Ghrelin, Glucose-dependent Insulinotropic Polypeptide (GIP), Glucagon-like peptide-1 (GLP-1), and Adiponectin] were measured using Bio-Plex Pro Human Diabetes Assay panel, Bio-Rad, following the manufacturer’s protocol^18^. A detailed protocol is provided with supplementary materials.

### Plasma sample preparation and metabolomics analysis

An Ultra-Performance Liquid Chromatography, ACQUITY UPLC System (Waters) coupled to a Quadrupole-Time of Flight (Q-TOF) mass spectrometer (SYNAPT-G2 HDMS, Waters) was used for untargeted metabolomics analysis. An analytical batch comprised of equal number of samples from all the study groups and their run order was randomized within a batch. The Quality control samples were prepared by pooling equal volume of aliquots from all the samples. QC samples were analyzed after every 5^th^ sample run. The features detected in <50% of the QC samples and <20% of the experimental samples were removed to exclude metabolites with poor repeatability in the metabolomics data. After normalization, features with a relative standard deviation of <30% in the QC samples were used for further statistical analysis.

Detailed sample preparation, liquid chromatography, and tandem mass spectrometry protocol, data transformations, and metabolite identification are provided with the supplementary materials.

### Statistical analysis

IBM SPSS Statistics-25, MetaboAnalyst, and R 3.2.5^19^ were used for statistical analysis, and GraphPad Prism 6.0 was used for graphical representations. Kolmogorov-Smirnov test and Shapiro-Wilk test were used for checking the normality of the data.

A one–way ANOVA was used to compare the clinical and biochemical parameters, HOMA-IR and OGIS values in NC, FDR, OW, and PRD groups. Tukey HSD post hoc test (p < 0.05) was used for multiple comparisons. Student’s t-test was used to compare HOMA-IR and OGIS values in men and women. Pearson’s correlation analysis was performed between the intensity of metabolites at basal level and OGIS values. Significantly changed markers of inflammation between groups (NC, FDR, OW, and PRD) were identified by the ANCOVA method by selecting age and sex as the covariate. ANCOVA analysis was also performed to explore the level of metabolites changed in women and men by selecting age as a covariate. The estimated marginal mean was used in plotting the figure. Bonferroni correction procedure was used for the correction of p-values.

Repeated measures ANOVA were used for three types of analysis in this study. Firstly, repeated measures ANOVA were done to explore the effect of time and risk factors on glucose, and insulin response, and the metabolite levels by selecting age and sex as covariates. Secondly, repeated measures ANOVA were performed to explore the effect of time and sex on glucose, insulin response, and metabolite levels by selecting age as a covariate. Bonferroni correction procedure was used to correct the p-values for multiple comparisons. A p-value of <0.05 was considered significant. Finally, the number of metabolites changed postprandially in each study groups such as NC, FDR, OW, and PRD was identified by repeated measures ANOVA analysis. Repeated measures ANOVA analysis of metabolomics data collected at 0 min, 60 min, and 120 min were performed with MetaboAnalyst software^20^, and False Discovery Rate (FDR) procedure was used for the p-value correction.

Principal Component Analysis (PCA) and Partial Least Squares Discriminant Analysis (PLS-DA) were performed by MetaboAnalyst software to identify the class differences from a multivariate metabolomics dataset^20^. Sum normalization method was used to correct the metabolite peak intensity unrelated to biological difference due to small variations in sample preparation and performance of the instrument. Log transformation was used to correct the skewed distribution and heteroscedasticity present in the metabolomics dataset^21^.

We created a linear mixed effect model for outcome variable OGIS using the GAD package in R 3.2.5. ‘Group’ (which indicated NC, FDR, OW or PRD) was treated as a fixed effect predictor since we had picked subjects belonging to the four groups on purpose; ‘Sex’ was treated as random within ‘Group.’ The other predictor in the model was ‘Age’ as a continuous variable. The predicted values were charted using ‘ggplot2’ package in R.

## Results

### Baseline characteristics of study subjects

**Figure 1:**
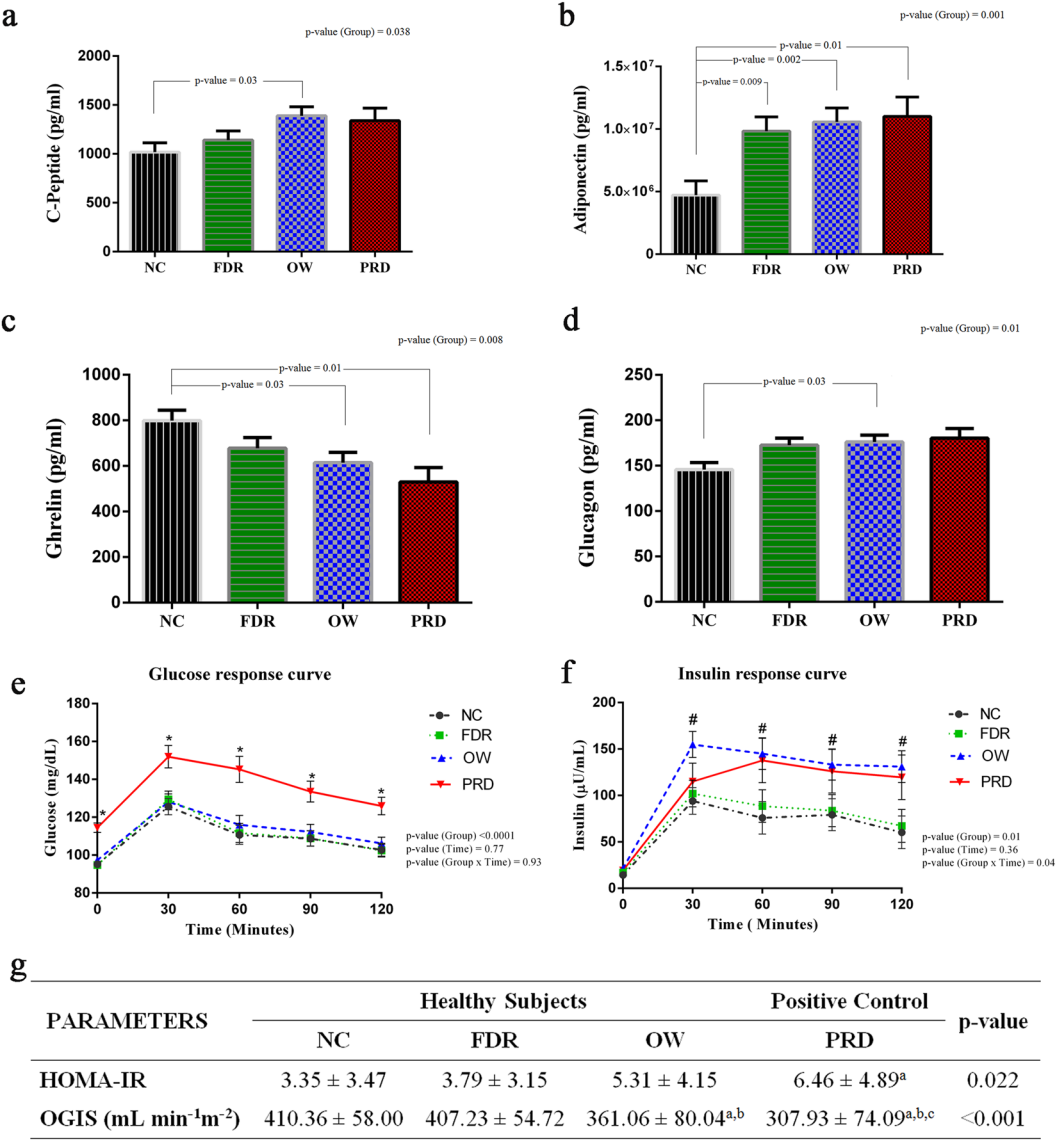
Effects of Family History of T2DM, Overweight, and Prediabetes on Insulin Sensitivity and Markers for Inflammation and T2DM. (**a**–**d**) Markers for inflammation and T2DM in each group. ANCOVA analysis was performed to explore the level of risk markers in NC, FDR, OW, and PRD groups by selecting age and sex as covariates. A p-value of <0.05 was considered significant by applying Bonferroni correction procedure for multiple comparisons. The estimated marginal means (EMM) adjusted for age and sex were used for representation. (FDR- First-Degree Relatives of patients with T2DM, OW - Overweight, PRD - Prediabetes, and NC - Normal Healthy Control). (**e**,**f**) Repeated measures ANOVA were performed to explore the effect of time and risk factors on glucose and insulin response to a mixed-meal challenge. Symbols indicate significant difference from NC subjects compared to PRD (*) and OW (#). (**g**) A one-way ANOVA was conducted to compare the HOMA-IR and OGIS values in healthy groups such as NC, FDR, OW, and PRD group. Tukey HSD post hoc test (p < 0.05) was used for multiple comparisons (a-significantly different from NC, b- significantly different from FDR, and c- significantly different from OW).

The basal clinical and biochemical profile of study subjects in four groups such as NC, FDR, OW, and PRD are given in Supplementary Table S2. Subjects with prediabetes were different in age and had dissimilar BMI, WHR, FBS, and HbA1c (%) compared to the subjects of the other three study groups. CRP and HDL cholesterol levels were significantly altered in OW subjects compared to the NC group. Serum levels of C-Peptide, and glucagon were significantly elevated in the OW group (Fig. 1a,d). Adiponectin level was significantly elevated in both OW and PRD groups (Fig. 1b). Ghrelin, also known as the hunger hormone, was significantly reduced in the OW and PRD groups (Fig. 1c). Adiponectin was the only marker altered in the subjects of the FDR group compared to the NC group (Fig. 1b**)**. Thus, the baseline profile was similar in NC and FDR group while overweight and prediabetes subjects had a dissimilar profile compared to the normal controls (Fig. 1a–d**)**.

### Insulin sensitivity is reduced in overweight healthy individuals

All study participants were subjected to a mixed-meal challenge to study the differences in postprandial metabolism. The plasma levels of glucose and insulin at 30 minutes intervals for two hours after the mixed-meal intervention are plotted in Fig. 1e,f. Repeated measures ANOVA revealed that glucose and insulin responses were significantly different between groups. At all 30 minutes’ intervals, the level of plasma glucose was elevated in the PRD group. Even though subjects who are overweight had similar levels of glucose as those in the NC group, they had significantly higher levels of insulin at all time intervals as in subjects of the PRD group. There was no difference in HOMA-IR (fasting blood sugar-based insulin sensitivity) between the OW group and the FDR and NC groups. However, OGIS, based on postprandial glucose values was significantly reduced in the OW group compared to NC and FDR groups (Fig. 1g**)**; OGIS was reduced significantly in the PRD group as well. Postprandial responses of insulin in overweight subjects indicated insulin resistance in them though they had fasting and postprandial blood sugar levels similar to the NC group **(**Fig. 1e,f**)**.

### Postprandial metabolism is different in overweight, and prediabetes subjects

**Figure 2:**
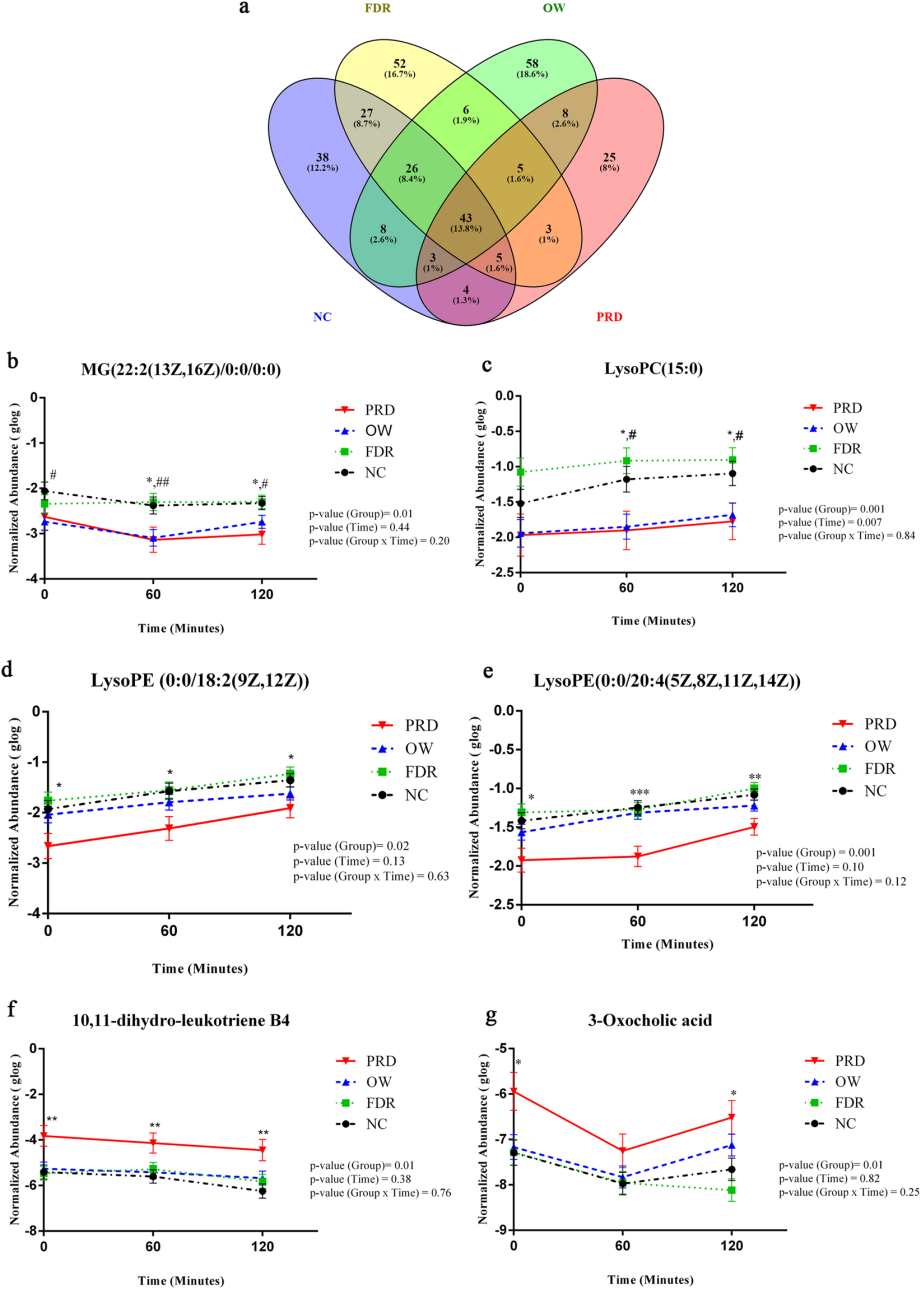
Postprandial Metabolism is Different in Overweight individuals and Those with Prediabetes. (**a**)The metabolites changed significantly at 0 min, 60 min, and 120 min in NC, FDR, OW, and PRD groups were identified by repeated measures ANOVA analysis. Repeated measures ANOVA analysis was performed with MetaboAnalyst software and False Discovery Rate <0.05 was considered as significant. (**b**–**g**) Metabolic alterations between study groups at 0 min, 60 min, and 120 min were identified by repeated measures ANOVA. Symbols indicate significant difference from NC subjects compared to PRD (*), OW (#), and FDR ($) by post hoc comparisons. A p-value of <0.05 was considered as significant by applying Bonferroni correction procedure for multiple comparisons. Error bar represents standard error (SE).

Plasma samples collected at 0 min, 60 min, and 120 min were analyzed by untargeted metabolomics to explore the postprandial metabolism. A signal correction was performed with the QC-RLSC method to correct batch influence (Supplementary Fig. S1). After QC-RLSC normalization, 3745 features with a relative standard deviation of <30% in the QC samples were considered for further statistical analysis. Of these, 1253 features were annotated (Supplementary Table S3). Repeated measures ANOVA analysis indicated that the numbers of metabolites that changed postprandially in subjects of the PRD group (Total-96, Annotated-54 and Unannotated-42) were less compared to the numbers of metabolites that changed in subjects of the OW group (Total-157, Annotated-66 and Unannotated-91), FDR group (Total-167, Annotated-77 and Unannotated-90) and the NC group (Total-154, Annotated-90 and Unannotated-64) groups (Fig. 2a). Metabolites such as MG(22:2(13Z,16Z)/0:0/0:0) and LysoPC (15:0) were significantly reduced in overweight subjects when compared to subjects of NC and FDR groups. LysoPE (0:0/18:2(9Z, 12Z)), LysoPE (0:0/20:4(5Z, 8Z, 11Z, 14Z)), 10, 11-dihydro-leukotriene B4, and 3-Oxocholic acid were significantly altered in the subjects of the PRD group, with a different pattern compared to the NC group; however the changes were not statistically significant in the OW group (Fig. 2b–g). These results suggest postprandial metabolic responses after a mixed-meal challenge are different in overweight individuals.

### Sex differences in insulin sensitivity and metabolome

**Figure 3:**
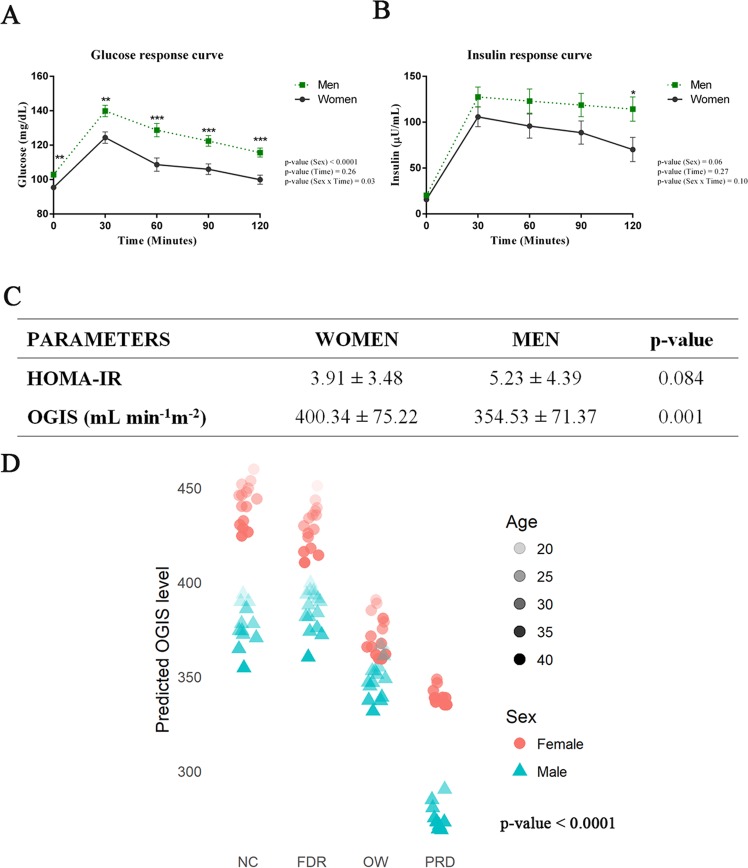
Men Have Reduced Insulin Sensitivity. **(a)** Postprandial Plasma Glucose Level. **(b)** Postprandial Plasma Insulin Level. Repeated measures ANOVA were performed to explore the effect of time and sex on glucose and insulin response to a mixed meal challenge. Asterisks (*) indicate a significant difference between sexes by post hoc comparisons. P-values were corrected by applying the Bonferroni correction procedure for multiple comparisons. (**c**) Student’s t-test (p-value <0.05) was used to compare the HOMA-IR and OGIS values in men and women. Error bar represents standard error (SE). (**d**) Linear mixed effect model for outcome variable OGIS predicted by using the GAD package in R (adjusted R^2^ = 0.33; p-value <0.0001).

**Figure 4:**
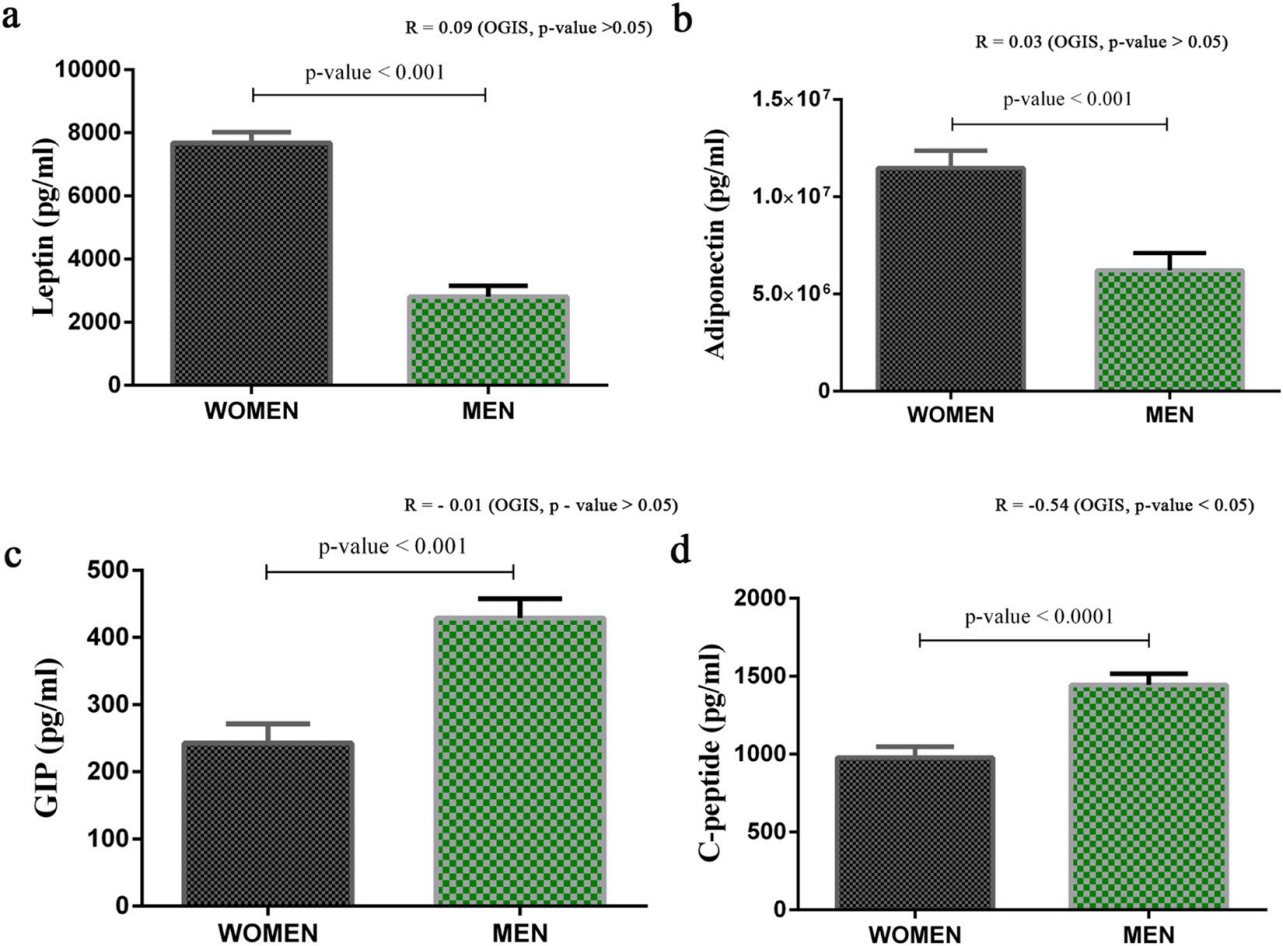
Effects of Sex on Markers for Inflammation and T2DM. (**a**–**d**) Comparison of markers for inflammation and T2DM in men (n = 55) and women (n = 55). ANCOVA analysis was performed to explore the level of risk markers in women and men by selecting age as a covariate. Estimated marginal means (EMM) adjusted for age was used for representation. Error bar represents standard error (SE). P-values were reported based on ANCOVA after adjusting age. Correlation coefficients and p-values from ANCOVA analysis are labeled in the figure and correlation coefficient values are provided in Supplementary Table S5.

Repeated measures ANOVA analysis revealed that men and women had a difference in postprandial glucose response (Fig. 3a). An elevated level of plasma glucose was observed in men at each time points. Insulin levels were also elevated at each time point in men, though the difference was not significant (Fig. 3b). Insulin sensitivity indices, including HOMA-IR and OGIS, indicated a significantly reduced level of insulin sensitivity in men (Fig. 3c**)**. A linear mixed effect model analysis predicted males to have lower expected values of OGIS than females in all the groups, and the values decreased with age (Fig. 3d**)**. The difference in insulin sensitivity between men and women observed in all the study groups led us to explore the sex differences in insulin sensitivity and metabolome further. The baseline levels of triglycerides (R = −0.360, p-value <0.0001) and VLDL (R = −0.361, p-value <0.0001), which are negatively correlated with insulin sensitivity, were elevated in men. A reduced level of HDL cholesterol was observed in men, and it was positively associated (R = 0.26, p-value = 0.007) with insulin sensitivity (Supplementary Tables S4 and S5). Levels of leptin (R = 0.09, p-value = 0.30) and adiponectin (R = 0.03, p-value = 0.75) were significantly higher in women (Fig. 4a,b) and these hormones may have a positive influence on insulin sensitivity in women. GIP (R = −0.01, p-value = 0.87) and C-peptide (R = −0.54, p-value <0.001) markers associated with insulin resistance were significantly elevated in men (Fig. 4c,d and Supplementary Table S5**)**.

### Reduced insulin sensitivity correlates with the metabolome in men

**Figure 5:**
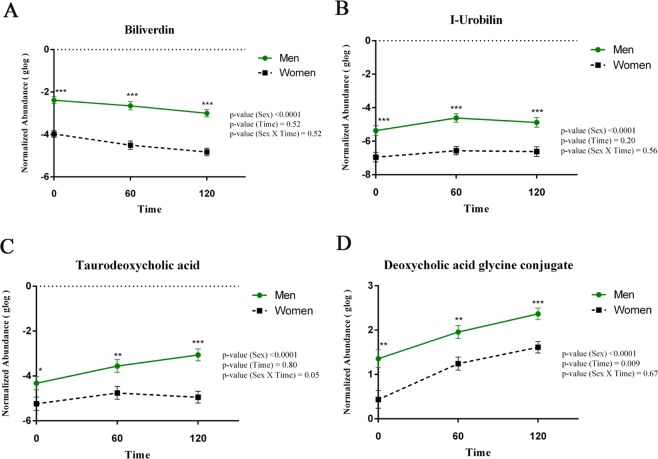
The Postprandial Bile Secretion is Distinct in Men and Women. (**a**–**d**) Repeated measures ANOVA were used to identify significantly altered bile acids between men and women at 3-time points. Asterisks (*) indicate a significant difference determined by post hoc comparisons. A p-value of <0.05 was considered significant by applying Bonferroni correction procedure for multiple comparisons. Error bar represents standard error (SE).

The influence of sex on the metabolite profile was checked by a supervised multivariate Partial Least Squares-Discriminant (PLS-DA) analysis using MetaboAnalyst. In PLS-DA analysis, metabolomics data with respect to the first five components were found to be different in men and women subjects (Supplementary Fig. S2). Metabolites such as biliverdin (Fig. 5a**)**, I-Urobilin (Fig. 5b**)**, taurodeoxycholic acid (Fig. 5c**)**, and deoxycholic acid glycine conjugate, all metabolites related to bile acid secretion (Fig. 5d**)** were significantly elevated in men.

**Figure 6:**
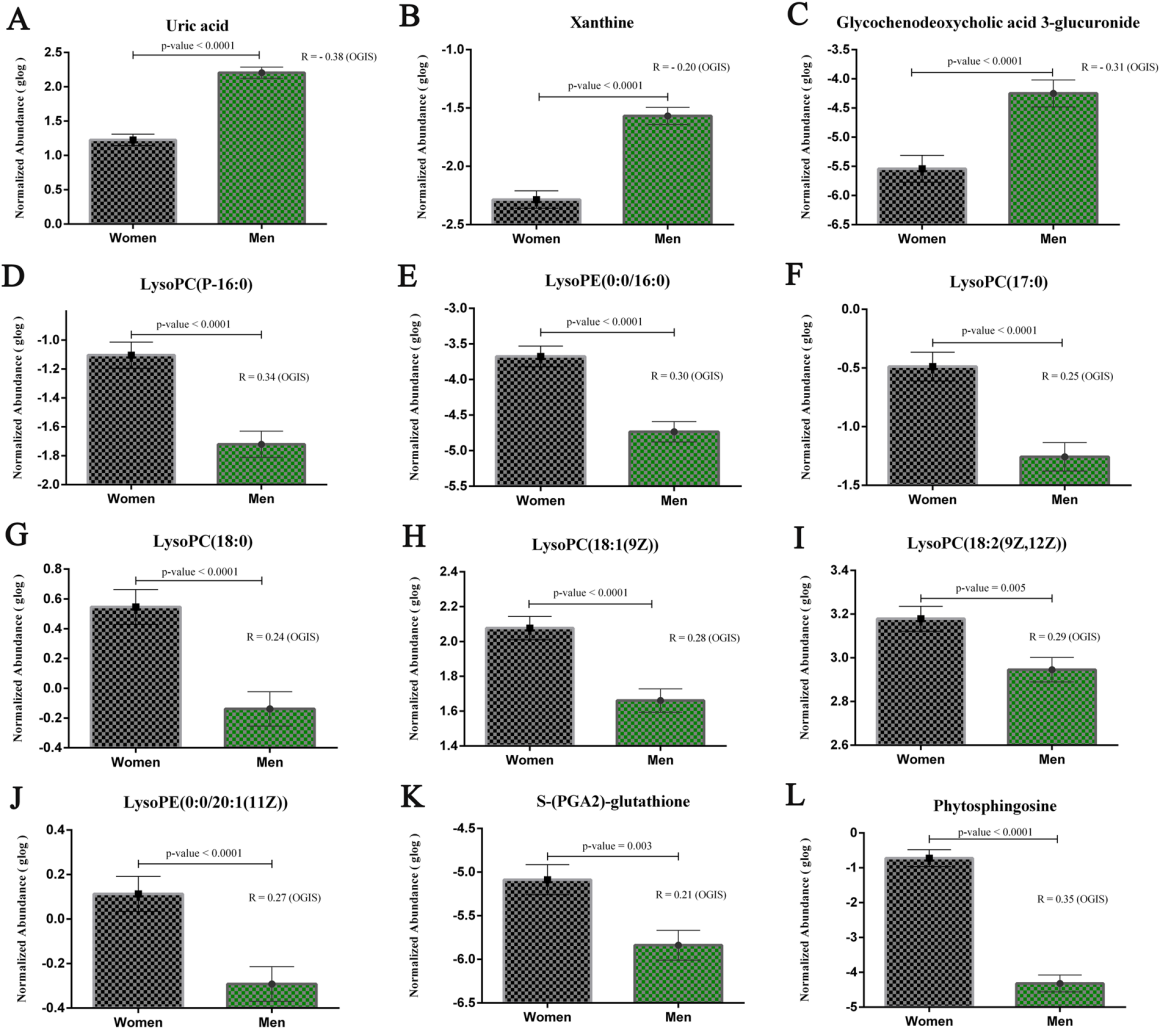
Metabolites Correlated with Insulin Sensitivity. (**a**–**l)** Pearson’s correlation analysis was performed between the intensity of metabolites at basal level and OGIS with corrplot package implemented in R 3.2.5. A correlation coefficient with a p-value of <0.05 was considered as significant. Estimated marginal means (EMM) adjusted for age was used for representation. Error bar represents standard error (SE). P-values were reported based on ANCOVA after adjusting age. Correlation coefficients and p-values from ANCOVA analysis are labeled in the figure and correlation coefficient values are provided in Supplementary Table S5.

Pearson’s correlation analysis was performed between the intensity of metabolites at basal level and OGIS values. ANCOVA analysis was performed to explore the level of metabolites in women and men by selecting age as a covariate (Fig. 6 and Supplementary Table S5). At fasting level, metabolites such as uric acid (Fig. 6a**)**, xanthine (Fig. 6b**)** and glycochenodeoxycholic-3-glucuronide (GCDC-3-glucuronide) (Fig. 6c) were significantly elevated in men and were negatively associated with OGIS. Metabolites including LysoPC (P-16:0) (Fig. 6d), LysoPE(0:0/16:0) (Fig. 6e**)**, LysoPC(17:0) (Fig. 6f**)**, LysoPC (18:0)(Fig. 6g**)**, LysoPC(18:1(9Z)) (Fig. 6h**)**, LysoPC(18:2(9Z,12Z)) (Fig. 6i**)**, LysoPE(0:0/20:1(11Z)) (Fig. 6j**)**, S-(PGA2)-glutathione (Fig. 6k**)**, and phytosphingosine (Fig. 6l**)** were elevated in women and were positively associated with OGIS. The results thus suggest a distinct postprandial metabolic profile that can be correlated with reduced insulin sensitivity in men.

**Figure 7:**
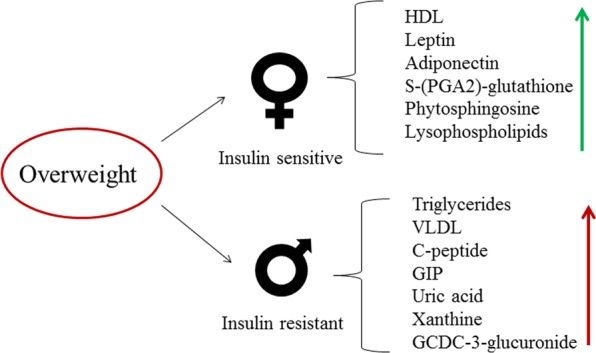
Effect of Overweight and Sex on Insulin Sensitivity and Metabolome.

In summary, our results reveal that overweight, but not yet obese, young adult men have increased insulin resistance though they have normoglycemia. Reduced insulin sensitivity observed in men was correlated with blood levels of triglycerides, VLDL, C-peptide, uric acid, xanthine, and GCDC-3-glucuronide (Supplementary Table S5). Elevated levels of HDL, leptin, adiponectin, glutathione-conjugate, phytosphingosine and lysophospholipids appear to protect young women from insulin resistance (Fig. 7).

## Discussion

Our study reveals early metabolic changes associated with insulin resistance in normoglycemic young adults who are overweight but not yet obese. Overweight subjects were found to have reduced insulin sensitivity. Serum lipid profile and obesity/diabetes related markers were altered, and postprandial metabolic signatures were distinct in overweight subjects. Altered levels of CRP and HDL-cholesterol as reported in obese individuals were seen in overweight subjects^22,23^.

A relatively higher degree of insulin resistance was found in young adult men compared to young adult women. We also found differences between young men and women in the levels of leptin, adiponectin, GIP and C-peptide, and postprandial changes in bile acid metabolites; these differences may account for the disparity in insulin sensitivity seen between sexes. Blood levels of metabolites related to purine metabolism, lysophospholipids, and antioxidants were altered in men and found to correlate with insulin sensitivity. Together, our findings indicate that overweight young adults have factors that make them prone to develop hyperglycemia. They had insulin resistance and elevated blood levels of important obesity/diabetes markers as in young adults with prediabetes but had normal fasting blood glucose levels. Interestingly in the same age group, those with a family history of diabetes did not have these features.

A large number of metabolomics studies have been conducted in patients with T2DM^24–28^. Metabolomics analysis has, however, not been widely used to identify altered metabolism in healthy people who are at risk for T2DM^6,29^. A Chinese cohort study and PREDIMED trial had employed targeted metabolomics^24,27^. A recent report of the PREDIMED Trial study indicates a non-targeted metabolomics approach. This investigation found that plasma metabolites predict insulin resistance and incident T2DM^26^. Metabolite profile of individuals with normal fasting glucose is also available in this report. A recent prospective cohort study in South African women indicated that changes in the metabolism of phospholipids, bile acids, and branched-chain amino acids predict the development of T2DM^30^.

The difference in our study from previous studies is that our subjects included those with a body mass index suggesting overweight but not obesity, those who had first degree relatives with T2DM and those with prediabetes, all thus having normoglycemia but known to have the risk to develop T2DM. The role of obesity (BMI >30 kg/m^2^) in the pathogenesis of T2DM is well recognized^31^. Overweight during childhood and adolescence is an independent risk factor for the development of T2DM in young adults^32^. First degree relatives of patients with T2DM are well known to have an increased risk for T2DM^33^. Significantly, our study reveals that healthy normoglycemic young adults who are overweight but not obese could have increased insulin resistance as seen in those with prediabetes. Fasting serum levels of C-peptide, an indirect measure of insulin secretion from pancreatic β-cells and glucagon were also altered in overweight subjects corroborating the presence of insulin resistance in overweight subjects^34^. Insulin resistance, atherosclerosis, and dyslipidemia are known to be associated with a lower level of adiponectin^35^. Higher levels of adiponectin were observed in overweight subjects of our study and maybe because of adiponectin resistance as has been reported in obese individuals by others^36^. In our study, young adults with overweight had reduced levels of ghrelin. Ghrelin stimulates appetite and regulates energy balance. Ghrelin concentrations are reduced in different pathophysiological conditions, including obesity and T2DM^37^.

The progressive development of T2DM is associated with weakening of metabolic flexibility and alterations in the capacity to adapt to external metabolic challenges such as diet^38^. Dietary challenge tests can reveal regulatory processes associated with metabolic flexibility^39^. Dietary challenge tests are useful to determine the capacity of an individual to adapt to metabolic challenges and dynamic alterations in nutrient metabolism^40^. The dietary challenge tests such as oral glucose tolerance test (OGTT), oral lipid tolerance test (OLTT), and oral protein tolerance test (OPTT) are used to investigate the flexibility of glucose, lipid and protein metabolism respectively^41,42^.

We used a mixed-meal challenge test to evaluate the postprandial metabolic alterations in young adults. A mixed-meal challenge, in contrast to an isolated nutrient, can target multiple organs and could evoke a normal physiological response^43^. A mixed-meal challenge test is considered superior to the oral glucose tolerance test as the former induces postprandial hyper triglyceridemia as well as hyperglycemia^44^. To our knowledge, only three studies have been conducted in those at risk for T2DM, to discover metabolomic changes after a mixed-meal challenge. Altered levels of oxylipins profiles after a high-fat meal was reported in obese individuals compared to lean subjects in a targeted analysis^45^. A targeted study identified altered levels of serine, glycine, acylcarnitines, and lysophosphatidylcholines in people with impaired fasting glycemia and T2DM^46^. Transcription factor 7-like 2 (TCF7L2) genes are one of the strongest genetic predictors for T2DM. When non-diabetic men carrying the high-risk homozygous genotype (TCF7L2) was subjected to a mixed-meal challenge test, in an untargeted metabolomics analysis, alterations were found in postprandial phospholipid metabolism^47^.

Postprandial hyperinsulinemia was observed in overweight subjects of our study, clearly indicating underlying insulin resistance. Interestingly, in response to the meal challenge, subjects with prediabetes had changes in lesser number of metabolites in comparison with overweight subjects and controls. The lack of marked alterations in metabolites could indicate a sluggish metabolism in subjects with prediabetes; this may also be because of an altered level of metabolites at the resting fasting state or postprandial hyperinsulinemia and insulin resistance in individuals with prediabetes^44^. Framingham heart study reported that fluctuations in the levels of branched chain amino acids and aromatic amino acids are associated with changes in body mass index. We did not find significant alterations in the levels of these amino acids after the mixed-meal intervention in our subjects. After the mixed-meal challenge, mostly lipids such as lysophospholipids were significantly altered in the PRD and overweight groups. Previous studies reported that lysophospholipids could act as lipokines and can play an essential role in insulin secretion and peripheral insulin sensitivity^48,49^. 10, 11-dihydro-leukotriene B4, a key player in low-grade inflammation, promoting insulin resistance^50^ was also found altered among PRD groups in our study.

Another significant finding of our study is the prominent role of biological sex in metabolism. In a recent study on a cohort of 184 consecutive patients with diabetes, we found that 78% of patients with early onset (age 20–45 years) of diabetes and 53% of patients with normal onset T2DM (age >50 years) were men^51^. In our present study also, postprandial hyperglycemia was preponderant in healthy men compared to women. Measures of insulin sensitivity indicated insulin resistance in men, and insulin resistance was associated with alterations in the lipid profile and elevated serum levels of C-peptide and GIP^18^, which are known to be associated with insulin resistance. The levels of two key adipokines such as leptin and adiponectin were observed to be elevated in women. Leptin is known to improve peripheral insulin sensitivity and pancreatic β-cell function^52^. The insulin-sensitizing action of adiponectin is from the decrease in hepatic gluconeogenesis and increase in muscle glucose transport^53^.

We found that bile acid metabolism was altered differently in men and women. Bile acids are involved in lipid and glucose metabolism by activating different signaling pathways such as membrane receptor TGR5 and the nuclear receptor farnesoid X receptor (FXR). Dysregulations in bile acid homeostasis have been implicated in the pathogenesis of T2DM^54^. Alteration of bile acids in response to oral glucose tolerance test (OGTT) was observed in metabolomics studies in healthy individuals, and these were associated with insulin sensitivity^8,55^. We hypothesize that the difference in postprandial bile acid levels in men may have a role in their reduced insulin sensitivity compared to women.

Several metabolites correlated with insulin sensitivity in both sexes at fasting state. Elevated levels of final oxidation products of purine metabolism such as uric acid and xanthine were observed in men and were negatively associated with OGIS index. Individuals, including young adults, with high serum uric acid, are at high risk of T2DM, independent of other known risk factors^56^. GCDC3-glucuronide is synthesized by UDP-glucuronyl transferase in the liver, and elevated levels were reported in T2DM patients^57^. Increased levels of GCDC3-glucuronide might have links to dysfunctional liver metabolism in men. Decreased levels of lysophospholipids were also observed in men and were positively associated with insulin sensitivity. A lower level of phytosphingosine positively correlated with insulin sensitivity was found in men. Therapeutic role of phytosphingosine has been explored since it activates PPARγ and enhances the insulin sensitivity^58^. Meal consumption has been shown to increase oxidative stress because of hyperglycemia and hyperlipidemia. The imbalance in postprandial antioxidant defenses can lead to the development of T2DM^44,59^. Increased level of hepatic glutathione in response to meal consumption has also been reported. Glutathione-conjugate such as S-(PGA2)-glutathione was positively associated with insulin sensitivity, and their levels were reduced in men. Though plasma samples collected at the fasting level, 60 minutes, and 120 minutes after the mixed-meal challenge were used for metabolomics analysis, most of the altered metabolites between the groups and between the sexes showed a significant difference at fasting level itself and that difference were maintained throughout. This property makes the identified metabolites as potential candidates for future biomarker studies without conducting a mixed-meal challenge test and postprandial measurements.

Our study indicates that when compared to young adult women, young adult men are more predisposed to T2DM. The risk for insulin resistance is higher in women during childhood and puberty. However, this is reversed in the adult stage, where men become more prone to insulin resistance^60,61^. This disparity is mainly because of the beneficial effect of estrogen on insulin sensitivity in women between the stage of puberty and menopause^62^. Sex differences in the metabolism begin at the early stages of development and continue after puberty. Metabolic dimorphism is determined by the type and number of sex chromosomes^63^, the masculinizing effect of perinatal testosterone surge from testis on the brain and liver, and the effect of female (17 β-estradiol, and progesterone) and male (dihydrotestosterone, and testosterone) sex hormones after the onset of puberty^64^. A previous targeted metabolomics analysis has shown that the concentrations of 102 out of 131 metabolites are significantly different between sexes^62^. A sex-specific metabolic difference was reported in the liver but not in the muscle or adipose tissue of mice. Liver maintains the synthesis of lipid molecules at the expense of amino acids during short-time fasting in females^65^. In a fasting state, liver in females tends to synthesize fatty acids and triglycerides and store them as fat. However, in males, fatty acid oxidation will be predominant in the fasting state. From an evolutionary perspective, energy storage will allow the woman to maintain the reproductive function during periods of limited food availability^62^.

During the onset of T2DM, unhealthy lifestyle factors such as high-calorie intake with low physical activity act as the metabolic load that surpass the metabolic capacity of an individual^66^. It is essential to maintain nutritional homeostasis in women at reproductive stages for the development of an embryo. Normal hepatic metabolism and sex hormones selected by evolution possibly protect women from metabolic disorders at the young adult stage; such mechanisms are absent in men.

Our study is unique as it is focused on apparently healthy young adults and is aimed at using untargeted metabolomics to identify early markers of risk to develop T2DM. This study has, however, some limitations. Targeted metabolomics analyses could have revealed metabolite identification and quantification specific to certain biochemical pathways. It remains unclear whether the changes in metabolites observed in our study, are contributory factors for insulin resistance or biomarkers of risk to develop T2DM. Less than 40% of the metabolites from our metabolome data could be annotated using available databases. Annotation and validation of those not annotated could provide novel insights for future studies. A mixed-meal challenge test can only give an approximation of results that could be obtained from the hyperinsulinemic-euglycemic clamp method. Inclusion of subjects with a family history of T2DM and overweight in our study could have helped to identify the combined effect of family history and overweight.

In summary, the present study provides new evidence for metabolic changes in young adults with risk for insulin resistance or T2DM and the basis for differential effects of those risks in men and women. Metabolic deviations from normal control individuals were identified in overweight young adults. Our study thus reveals that young adults with overweight, but not yet obese, are at risk for T2DM and the risk is more in men compared to women.

### Ethics statement

The study protocol was approved by the Institutional Human Ethics Committee of Rajiv Gandhi Centre for Biotechnology. Informed consent was obtained from all the study participants in the study. This study was performed in accordance with the Declaration of Helsinki.

## Supplementary information

Supplementary Information.

## Data Availability

Metabolomics data have been submitted to MetaboLights (Study ID: MTBLS743). All the authors confirm the availability of data and materials upon request.
