# Supplementary material for: Postprandial Metabolism is Impaired in Overweight Normoglycemic Young Adults without Family History of Diabetes
Source: Sci Rep. 2020 Jan 15;10:353. doi: 10.1038/s41598-019-57257-2 (PMC6962374; doi:10.1038/s41598-019-57257-2)
Supplement: Supplementary file 1 — Supplementary Information. [file 41598_2019_57257_MOESM1_ESM.pdf]

Supplementary Information

Postprandial Metabolism is Impaired in Overweight Normoglycemic Young Adults without Family History of Diabetes

Aneesh Kumar A, Gopika Satheesh, Gadadharan Vijayakumar, Mahesh Chandran, Priya R Prabhu, Leena Simon, Vellappillil Raman Kutty, Chandrasekharan C Kartha, Abdul Jaleel

METHODS

Markers of Inflammation and T2DM

Plasma markers of inflammation and T2DM [Interleukin 6 (IL-6), C-peptide, Glucagon, Insulin, Leptin, Plasminogen Activator Inhibitor-1 (PAI-1), Resistin, Visfatin, Ghrelin, Glucose-dependent Insulinotropic Polypeptide (GIP), Glucagon-like peptide-1 (GLP-1), and Adiponectin] in the fasting state were measured using Bio-Plex Pro Human Diabetes Assay panel, Bio-Rad as per the manufacturer's protocol[1]. Briefly, the protocol was as follows: 50 µL of human plasma samples diluted in serum diluent (1:3) and various concentrations of each marker standards were added to a 96-well plate containing 50 µL of fluorescent antibody-conjugated beads. After 30 minutes of incubation at room temperature, the plate was washed, 25 µL of a biotinylated detection antibody solution was added to each well and incubated again at room temperature for 30 min. After washing, 50 µL of streptavidin-conjugated phycoerythrin was added to each well and incubated for another 10 min at room temperature. Following a final wash, the contents of each well were resuspended in 125 µL of assay buffer and analyzed using a Bio-Plex Suspension Array Reader (Bioplex-200 system, Bio-Rad). The concentrations of each marker in the plasma samples were calculated from the standard curve of each marker standards. The inter- and intra-assay variability were <15 % and <10% respectively.

Plasma Sample Preparation and Metabolomics Analysis

Plasma samples stored at -80°C were thawed in ice, then vortexed, and centrifuged at1620g for 15 min at 4° C. For protein precipitation, 100 µL of each sample was mixed with 400 µL methanol and incubated at -20° C. After 1 hour (h), samples were vortexed for 1 min and

centrifuged at 14000g at 4° C for 20 min. The supernatant from each tube was collected and dried at room temperature using the SpeedVac concentrator. Dried samples were stored at -80°C until the analysis. During analysis, dried samples were dissolved in 100 µL of reconstitution solvent containing 50% methanol and 0.1% formic acid. The Quality control samples were prepared by pooling equal volume (600 µL) of aliquots from all the samples and processed as described above.

Liquid Chromatography and Tandem Mass Spectrometry

An Ultra-Performance Liquid Chromatography, ACQUITY UPLC System (Waters) coupled to a Quadrupole-Time of Flight (Q-TOF) mass spectrometer (SYNAPT-G2HDMS, Waters) was used for the analysis. Reversed-Phase Liquid Chromatography (RPLC) technique was employed with a C18 column (ACQUITY UPLC HSS T3 C18 column, 100 A$^{\circ}$, 1.8 µm, 2.1mm X 100mm, Waters). In the RPLC Method, mobile phase consisted of aqueous (A) and organic (B) solvent components, where A was 0.1% formic acid in water and B was 0.1% formic acid in methanol. The gradient was 0 min, 0% B; 1 min, 0% B; 16 min, 100% B; 20 min, 100% B, and 22 min, 0% B. The curve parameter of 5 was used throughout the gradient from 0 to 20 min and a curve parameter of 1 was used from 20 to 22 min. The same gradient was used for both positive and negative ion mode methods. For the RPLC wash cycle, weak wash solvent (100% water) and strong wash solvent (100% methanol) were used. Similarly, the Hydrophilic Interaction Chromatography (HILIC) method was performed using a BEH column (ACQUITY BEH HILIC column, 130 A$^{\circ}$, 1.7 µm, 2.1mm X 150mm, Waters). For the HILIC mobile phase, A was 95% Acetonitrile in 10mM ammonium acetate, and B was 50% Acetonitrile in 10mM ammonium acetate. Both A and B contained 0.1% formic acid and 0.1% acetic acid in positive ion mode and negative ion mode, respectively. The HILIC positive ion gradient was 0 min, 1% B; 5 min, 20%B; 10 min, 50% B; 14 min, 95%B; 16 min, 95% B; 17 min, 1%B; and 20 min, 1%B. The curve parameter of 6 was used throughout the gradient. The HILIC negative ion gradient was 0 min, 1% B; 3 min, 20% B; 6 min, 50% B; 9 min, 95%B; 10 min, 95% B; 11 min, 1% B; and 15 min, 1% B. The curve parameter of 6 was used throughout the gradient. For the HILIC wash cycle, weak wash solvent (100% acetonitrile) and strong solvent (100% water) were used. The injection volume was 5 µL with a flow rate of 0.4mL/min, and the column temperature of 40°C.

The mass spectrometer was operated in resolution mode (MS^E) with electrospray ionization (ESI). Data were acquired in both positive and negative modes for the two chromatographic methods. The selected mass range was from 50 to 1200 m/z in full scan mode with a scan time of 0.25s and an inter-scan delay of 0.024s. Ion source and desolvation gas (nitrogen) temperatures were kept at 112°C and 350°C respectively. The sampling cone and desolvation gas flow rates were 60 L/h and 875 L/h, respectively. The capillary voltage was set at 2.85 kV. Sampling cone voltage was set at 40V. The *lock mass* acquisition was made every 10s by Leucine enkephalin (MW= 555.62) for accurate on-line mass calibration. Initial ten QC samples run was used for the stabilization of the system (~4h). QC samples were analyzed after every 5th sample run. An analytical batch comprised of an equal number of samples from all the study groups and their run order was randomized within a batch. One analytical batch had 90 runs, and data were acquired up to 35h without affecting the stability of the system.

Data Transformation

MassLynx4.1 SCN781 (Waters) was used for data acquisition and collection. Progenesis QI (Non-linear dynamics, Waters) was employed for peak/feature picking and raw data deconvolution. Progenesis QI performed noise filtering, peak detection, isotope peak removal, alignment of retention time and mass, as well as optional peak/feature normalization. Progenesis produced a feature matrix that contained accurate mass (m/z), retention time (RT), and chromatographic peak area. MetaX software was used for further processing of the normalized data[2]. The features detected in <50% of the QC samples and <20% of the experimental samples were removed to exclude metabolites with poor repeatability in the metabolomics data[2,3]. The missing values retained after the initial filtration were imputed with the k-nearest neighbor method. A signal correction was performed with the QC-RLSC (Quality Control-Robust Loess Signal Correction) method to correct the batch influence. After normalization, features with a relative standard deviation of <30% in the QC samples were used for further statistical analysis[3].

Metabolite Identification

Progenesis MetaScope (Waters, Nonlinear Dynamics, USA) was used for the putative annotation of metabolite features. The annotation was based on accurate mass values, isotope abundance, and MS/MS fragmentation patterns. Metabolites identification search was performed within a

narrow mass error (10 ppm) by selecting databases such as the Human Metabolome Database (HMDB2016) and MassBank of North America (MoNA). MS/MS fragmentation matching patterns were also evaluated, and those annotations matched with fragmentation database were labeled as MS level 2, and remaining annotations were labeled as MS Level 3[4]. Metabolomics data were submitted in MetaboLights (Study ID: **MTBLS743**).

Supplementary Table S1: Representative table showing the composition of mixed-meal (Breakfast). A representative female study subject (Moderate lifestyle) with an ideal body weight of 54 kg requires approximately 1890 kcal per day according to Recommended Dietary Allowances (RDA) for Indians (ICMR, 2010)[5]. A mixed-meal with ~473 Kcal (25% of the Total Calorie requirement) was used for the tolerance test in this study subject. The calorie contents of each food item were calculated from the data published for Indian foods by the National Institute of Nutrition, Indian Council of Medical Research[6].

Item	Amount	Energy (Kcal)	Carbohydrate (g)	Fat (g)	Protein (g)
**Idli (rice cake)**	143 g	191.62	41.18	-	5.88
**Chutney (This sauce contains coconut and Bengal gram)**	120g	108.31	10.56	5.78	3.54
**Milk Tea**	150 ml	100.5	6.6	6.15	4.8
**Skimmed milk powder**	7 g	24.99	3.57	0.07	2.665
**Table sugar**	4 g	15.92	3.97	-	-
**Coconut oil**	3.5 ml	31.5	-	3.5	-
**Total**	-	**472.84**	**65.88**	**15.5**	**16.88**

Supplementary Table S2: Clinical and Biochemical Characteristics of Study Subjects. A one–way ANOVA was conducted to compare the parameters between the groups such as NC, FDR, OW, and PRD. Tukey HSD post hoc test ($p < 0.05$) was used for multiple comparisons (**a-** significantly different from NC, **b-** significantly different from FDR, and **c-** significantly different from OW). All data are expressed as the mean ± standard deviation.

PARAMETERS	Healthy Subjects			Positive Control	p-value
	NC	FDR	OW	PRD	
**Number**	30	30	30	20	1
**Age (Years)**	27.87 ± 5.72	27.73 ± 5.83	30.53 ± 5.62	36.95 ± 3.03^{abc}	<0.001
**BMI (kg/m^2)**	21.96 ± 1.78	22.28 ±1.98	27.23 ± 2.14^{a,b}	25.16 ± 3.16^{abc}	<0.001
**WHR**	0.83 ± 0.05	0.84 ± 0.04	0.89 ± 0.05^{a,b}	0.90 ± 0.06^{ab}	<0.001
**FBS (mg/dL)**	88.73 ± 8.44	87.50 ± 7.94	87.76 ± 9.61	107.05 ± 10.23^{abc}	<0.001
**HbA1c (%)**	5.35 ± 0.31	5.37 ± 0.28	5.39 ± 0.28	5.78 ± 0.40^{abc}	<0.001
**Total cholesterol (mg/dL)**	171.46 ± 35.23	169.90 ± 27.80	182.96 ± 27.10	188.65 ± 39.16	0.11
**Triglycerides (mg/dL)**	87.50 ± 46.22	84.20 ± 34.82	118.50 ± 90.87	113.45 ± 55.02	0.07
**HDL Cholesterol(mg/dL)**	38.76 ± 9.22	40.40 ± 11.79	32.83 ± 7.76^{b}	35.65 ± 6.57	0.01
**LDL Cholesterol (mg/dL)**	118.19 ± 24.99	112.30 ± 23.30	127.22 ± 24.39	130.04 ± 35.53	0.06
**VLDL (mg/dL)**	17.52 ± 9.26	16.94 ± 6.95	23.70 ± 18.17	22.69 ± 11.00	0.08
**CRP (mg/L)**	0.94 ± 1.20	1.92 ± 3.01	2.60 ± 1.91^{a}	1.64 ± 1.02	0.01

Supplementary Table S3: Overview of Mass Spectrometry Analysis of Metabolites.

Data Acquisition Method	Features	Annotated Features	Unknown Features
**RP- Positive**	1068	344	724
**RP-Negative**	1326	450	876
**HILIC – Positive**	622	200	422
**HILIC-Negative**	729	259	470

Supplementary Table S4: Clinical and Biochemical Characteristics of healthy men and women. Student t-test was used to compare two means and a p-value of <0.05 was considered significant. All data are expressed as the mean ± standard deviation.

PARAMETERS	WOMEN	MEN	p-value
**Number**	55	55	1
**Age (Years)**	31.51 ± 5.99	28.91 ± 6.33	0.038
**BMI (kg/m^2)**	23.97 ± 3.43	24.17 ± 2.88	0.593
**WHR**	0.85 ± 0.06	0.87 ± 0.05	0.047
**FBS (mg/dL)**	90.80 ± 11.67	92.12 ± 11.48	0.943
**HbA1c (%)**	5.50 ± 0.36	5.38 ± 0.33	0.037
**Total cholesterol (mg/dL)**	170.89 ± 32.12	183.70 ± 31.86	0.091
**Triglycerides (mg/dL)**	69.87 ± 31.15	129.67 ± 70.63	<0.001
**HDL Cholesterol(mg/dL)**	41.00 ± 10.09	33.05 ± 7.23	<0.001
**LDL Cholesterol (mg/dL)**	117.56 ± 24.74	124.84 ± 29.15	0.267
**VLDL (mg/dL)**	13.98 ± 6.22	25.99 ± 14.09	<0.0001
**CRP (mg/L)**	1.76 ± 1.68	1.82 ± 2.43	0.473

Supplementary Table S5: Biochemical parameters and metabolites associated with Insulin Sensitivity (OGIS) and Sex. Pearson's correlation analysis was performed between Biochemical parameters or the intensity of metabolites at basal level and OGIS with corrplot package implemented in R 3.2.5. A correlation coefficient with a p-value of <0.05 was considered significant. Student t-test or ANCOVA analysis was used to compare two means between sex and a p-value of <0.05 was considered significant. P-values were corrected by applying the Bonferroni correction procedure for multiple comparisons.

PARAMETERS	Pearson's correlation coefficient with OGIS	Sex (Up regulation)
**Triglycerides (mg/dL)**	- 0.36	Men
**HDL Cholesterol(mg/dL)**	0.26	Women
**VLDL (mg/dL)**	- 0.36	Men
**C-peptide**	-0.54	Men
**Uric acid**	-0.38	Men
**Xanthine**	-0.20	Men
**GCDC-3-glucuronide**	-0.31	Men
**LysoPC (P-16:0)**	0.30	Women
**LysoPC (17:0)**	0.25	Women
**LysoPC (18:1(9Z))**	0.28	Women
**LysoPC (18:1(9Z))**	0.29	Women
**S-(PGA2)-glutathione**	0.21	Women
**Phytosphingosine**	0.35	Women

SUPPLEMENTARY FIGURES

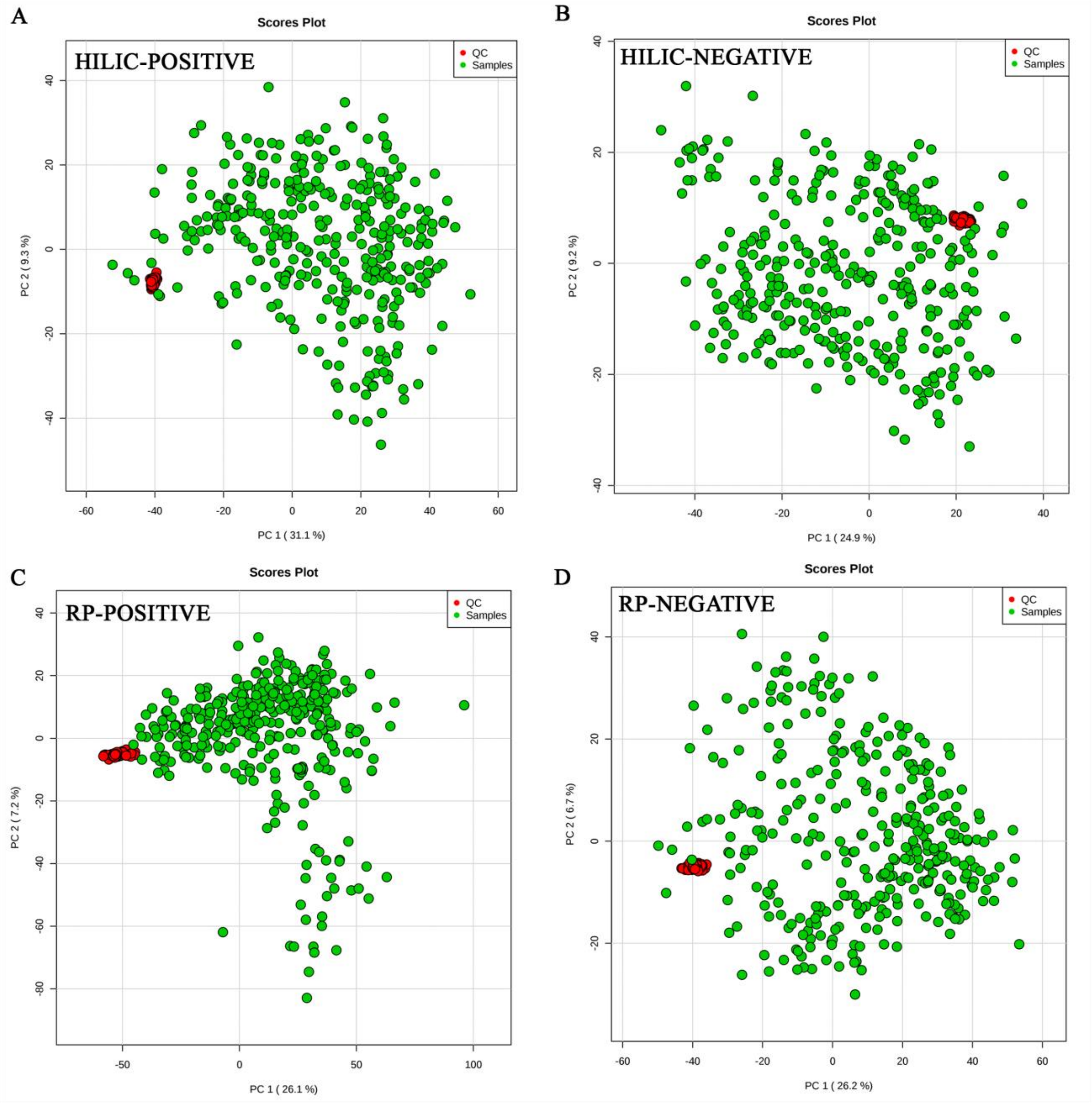

Supplementary Figure.S1: **Quality control PCA plots of UPLC-MS/MS data.** Principal Component Analysis (PCA) plots to show the analytical robustness of UPLC-MS/MS methods (**a-d**). Progenesis QI (Non-linear dynamics, Waters) was employed for peak/feature picking and raw data deconvolution. The missing values were imputed with the k-nearest neighbor method. A signal correction was performed with the QC-RLSC method to correct batch influence. MetaboAnalyst software was used for PCA analysis. Sum normalization method and log transformation were used for data transformation (QC- Quality Control samples).

PLS-DA Analysis

a

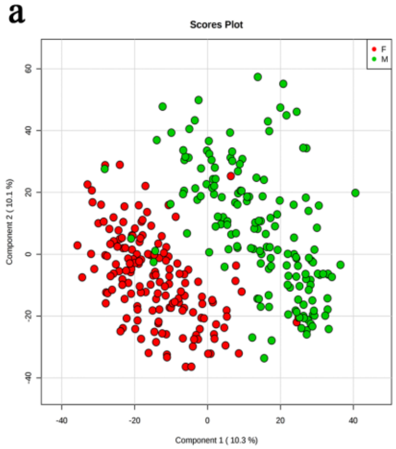

b

RP Positive

PLS-DA cross validation details:

Measure	1 comps	2 comps	3 comps	4 comps	5 comps
Accuracy	0.84949	0.95322	0.96271	0.96916	0.97842
R2	0.5786	0.74681	0.84741	0.89178	0.92199
Q2	0.54025	0.7083	0.80212	0.83987	0.86149

c

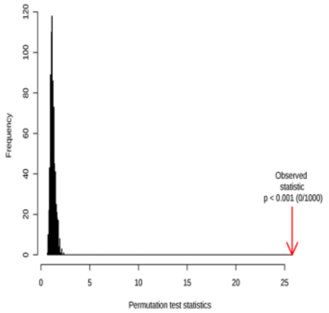

d

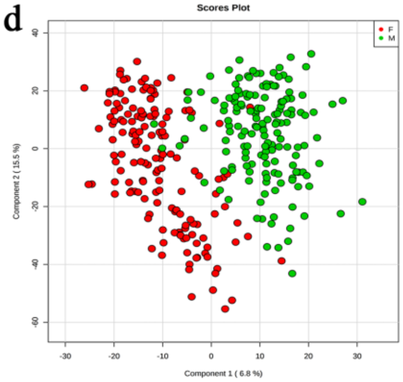

e

RP Negative

PLS-DA cross validation details:

Measure	1 comps	2 comps	3 comps	4 comps	5 comps
Accuracy	0.9059	0.93059	0.96293	0.96912	0.97864
R2	0.68623	0.76154	0.8507	0.89174	0.93002
Q2	0.628	0.71855	0.79016	0.81436	0.83929

f

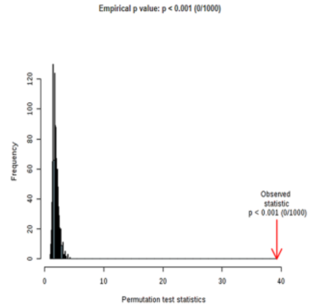

g

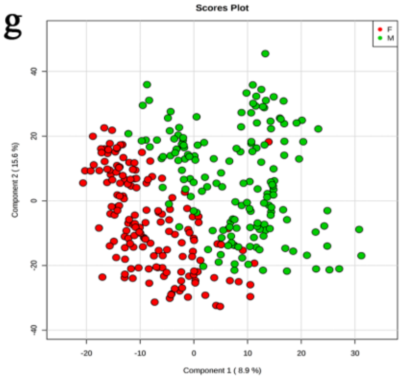

h

HILIC Positive

PLS-DA cross validation details:

Measure	1 comps	2 comps	3 comps	4 comps	5 comps
Accuracy	0.78722	0.89579	0.93147	0.94352	0.95015
R2	0.49639	0.64199	0.75842	0.826	0.863
Q2	0.43179	0.57792	0.69036	0.75041	0.7722

i

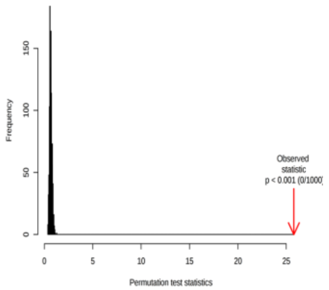

j

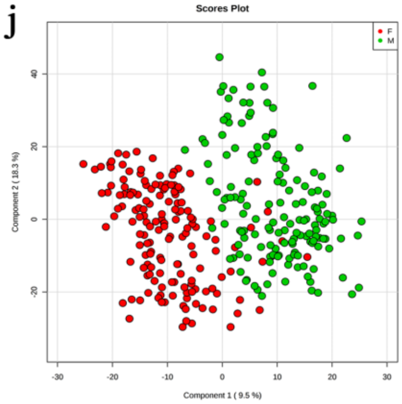

k

HILIC Negative

PLS-DA cross validation details:

Measure	1 comps	2 comps	3 comps	4 comps	5 comps
Accuracy	0.89813	0.94205	0.97103	0.97201	0.98099
R2	0.66674	0.73942	0.83634	0.88972	0.92629
Q2	0.63098	0.70528	0.79471	0.82566	0.85102

l

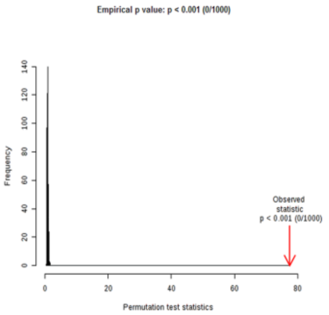

Supplementary Figure.S2: Postprandial metabolomics data can be classified by sex. PLS-DA analysis using MetaboAnalyst software represents the metabolomics data acquired in men and women by UPLC-MS/MS method (**a-l**). Sum normalization method and log transformation were used for the data transformation. PLS-DA model was evaluated by cross-validation of R^2 and Q^2 values with the first 5 components. A permutation test (permutation numbers-1000) was performed to test the statistical significance and p-value of <0.05 was considered significant.

REFERENCES

1. Vejrazkova, D. *et al.* Distinct response of fat and gastrointestinal tissue to glucose in gestational diabetes mellitus and polycystic ovary syndrome. *Physiol. Res.* **66,** 283–292 (2017).

2. Wen, B., Mei, Z., Zeng, C. & Liu, S. metaX: A flexible and comprehensive software for processing metabolomics data. *BMC Bioinformatics* **18,** (2017).

3. Dunn, W. B. *et al.* Procedures for large-scale metabolic profiling of serum and plasma using gas chromatography and liquid chromatography coupled to mass spectrometry. *Nat. Protoc.* **6,** 1060–1083 (2011).

4. Salek, R. M., Steinbeck, C., Viant, M. R., Goodacre, R. & Dunn, W. B. The role of reporting standards for metabolite annotation and identification in metabolomic studies. *Gigascience* **2,** (2013).

5. National Institute of Nutrition. Nutrient Requirements and Recommended Dietary Allowances for Indians a. *Rep. Expert Gr. Indian Counc. Med. Res.* 1–334 (2009).

6. Gopalan, C. *et al. Nutritional value of Indian foods. Hyderabad, National Institute of Nutrition* (2000).
